# Predictive value of the pathological extent of tumor invasion in endoscopic resection margins positive for residual tumor cells in surgically resected specimens of early gastric cancer

**DOI:** 10.3892/etm.2012.630

**Published:** 2012-07-03

**Authors:** HIRONORI TSUJIMOTO, SHO OGATA, YOSHIHISA YAGUCHI, ISAO KUMANO, RISA TAKAHATA, SATOSHI ONO, JUNJI YAMAMOTO, SHIGEAKI NAGAO, SOICHIRO MIURA, KAZUO HASE

**Affiliations:** 1 Departments of Surgery,; 2 Pathology and Laboratory Medicine and; 3 Internal Medicine, National Defense Medical College, Tokorozawa 359-8513, Japan

**Keywords:** early gastric cancer, incomplete endoscopic resection, radical gastrectomy

## Abstract

Although endoscopic resection (ER) is considered to be the optimal treatment for early gastric cancer, indications for radical gastrectomy in patients undergoing incomplete ER for early gastric cancer remain unclear. We evaluated the pathological extent of tumor invasion in the ER margins positive for residual tumor cells in the surgically resected specimens. We measured the vertical and/or horizontal length of the exposed tumor in the ER specimens of 23 patients with margins positive for tumor cells. We compared the clinicopathological data to distinguish between the presence and absence of residual tumor cells in the surgically resected specimens. Of 17 lesions with exposed tumor cells in the vertical margins of the ER specimens, only 3 (17.6%) had residual tumor cells in the corresponding site of the surgically resected specimens. By contrast, of 10 lesions with exposed tumor cells in the horizontal margins of the ER specimens, 8 (80.0%) had residual tumor cells in the corresponding site of the surgically resected specimens. The length of the exposed tumor in the vertical margins of the ER specimens was significantly associated with the incidence of residual tumor cells in the vertical margins of the surgically resected specimens. When the cut-off value for the length of the exposed tumor in the vertical ER margins was set to >3 mm, the sensitivity and specificity were 0.67 and 0.95, respectively. In conclusion, measurement of the length of the exposed tumor in the ER margins for early gastric cancer is a simple procedure that is able to determine whether additional surgical intervention is necessary.

## Introduction

Advances in diagnostic techniques have led to an increased incidence of small and early-stage gastric cancers (1,2). The incidence of early gastric cancer is >40% (3,4) and patients with early gastric cancer have an extremely favorable prognosis following curative treatment, with 5-year survival rates of >90% (4–6). The incidence of lymph node metastases originating from mucosal and submucosal lesions in early gastric cancer has been reported to be 3 and 20%, respectively (7); thus, standard gastrectomy with extensive lymphadenectomy may be inappropriate for such populations with regard to the quality of life (QOL) (8).

Endoscopic resection (ER), including endoscopic mucosal resection (EMR) and endoscopic submucosal dissection (ESD), may be the optimal treatment for early gastric cancer in terms of improving the QOL of the patient. However, ER has several problems; it occasionally fails to completely remove the cancerous lesion and pathological examination of the resected specimen may reveal a potentially high risk of lymph node metastases that does not meet the criteria for curative ER (9). Pathologists are occasionally unable to accurately evaluate the margin status following ER due to the burn effect and mechanical damage. Furthermore, even if exposed tumor cells are observed in the ER margin, residual tumor cells are not always detected in the surgically resected specimens (10,11). Thus, the selection of patients who require radical gastrectomy following incomplete ER for early gastric cancer is difficult.

In order to establish significant indications for radical gastrectomy in patients with ER margins positive for residual tumor cells, we reviewed the clinicopathological characteristics of patients who underwent incomplete ER for early gastric cancer. We also examined the predictive value of the pathological extent of the tumor invasion in the ER margins positive for residual tumor cells in the surgically resected specimens.

## Materials and methods

### Patients

Patients (n=758) with early gastric cancer underwent gastrectomy (n=207) or ESD (n=551) at the Departments of Surgery or Internal Medicine at the National Defense Medical College Hospital between 2005 and 2009. Since 2005, we have regarded the following features as indications for ESD according to the gastric cancer treatment guidelines in Japan (9): i) presence of differentiated-type carcinoma limited to the mucosal layer and ii) absence of ulceration or ulcer scars in the depressed type or irrespective of macroscopic type. A single-channel endoscope (GIF-H260; Olympus, Tokyo, Japan) was used in patients under conscious sedation. Lesions were marked beyond the margins using a conventional needle knife (needle papillotome; MTW Endoscopy, Wesel, Germany). A solution of 0.25% sodium hyaluronate in normal saline solution containing 0.001% epinephrine and 0.002% indigo carmine was injected into the submucosal layer and a circumferential incision was made to include the markings. Lesions were dissected using an insulation-tripped electrosurgical knife (EMR Knives; MTW Endoscopy) to curatively exfoliate tumors through the submucosal layer. ESD specimens were spread out, pinned on a flat cork and fixed in formalin solution. The size of the specimens, the size and shape of the tumor and the margins were recorded on a schematic diagram. Fixed materials were sectioned serially at 2-mm intervals parallel to a line that included the closest resection margin of the specimens (12).

**Figure 1:**
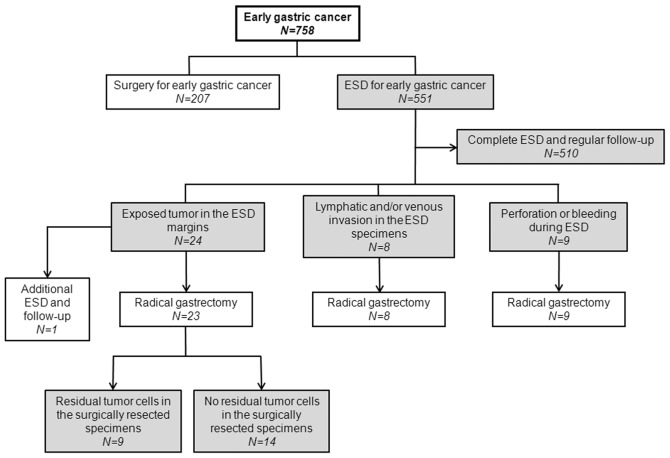
Flow chart showing the treatment outcomes in patients with early gastric cancer. ESD, endoscopic submucosal dissection.

Of the 551 patients who underwent ESD, 510 patients underwent complete ESD and regular follow-up. The remaining 41 patients underwent incomplete ESD, of which 40 underwent additional radical gastrectomy (Fig. 1). One patient whose ESD specimen showed exposed tumor cells in the vertical margin underwent additional ESD and intensive follow-up due to severe liver cirrhosis.

The clinicopathological findings of the patients were evaluated according to the Japanese Classification of Gastric Carcinoma (JCGC) published by the Japanese Gastric Cancer Association (12).

**Figure 2:**
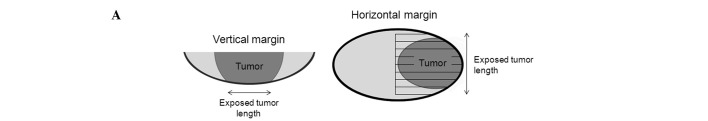

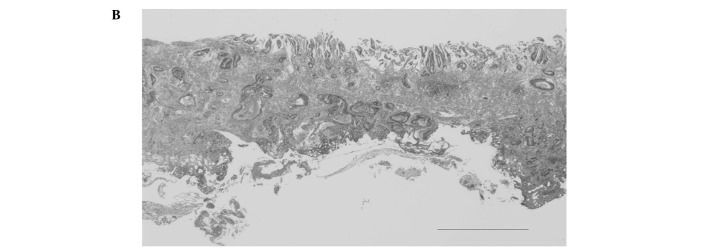
(A) Measurement of the length of the exposed tumor in the vertical and horizontal margins of ESD specimens. (B) Representative microscopic image (patient number 13 in Table II) for the measurement of the length of the exposed tumor in the vertical margins of ESD specimens. A scale expressing 1 mm is inserted. ESD, endoscopic submucosal dissection.

We measured the horizontal and/or vertical length of the exposed tumor in the ESD margins of 23 patients with ER margins positive for tumor cells. In the horizontal margin, we calculated the number of tumor-positive sections of a 2-mm width (horizontal lengths were calculated by the number of tumor-positive sections × 2; Fig. 2A). In the vertical margin, we microscopically measured the distance between the ends of the exposed tumor with a scale. The highest of these values was considered to be the vertical length of the exposed tumor. A representative microscopic image with a scale is shown in Fig. 2B. Pathological examination and measurement of the length in the ESD margins were evaluated by an author (S.O.), who was blinded to the pathological findings of the surgically resected specimens.

### Statistical analysis

The data are expressed as mean ± SD. The Mann-Whitney U test or the Chi-square test was used to compare the two groups. The ability of the clinicopathological data (including the length of the exposed tumor in the ESD margins, venous invasion and lymphatic invasion) to distinguish between the presence and absence of residual tumor cells in the surgically resected specimens was assessed using the area under the receiver operating characteristic (AUROC) curve. These data were analyzed using the MedCalc version 9 statistical software package (MedCalc software, Mariakerke, Belgium). P<0.05 was considered to indicate a statistically significant result.

## Results

Demographic data of the patients who underwent incomplete ESD for early gastric cancer are shown in Table I. The mean age of the patients who underwent incomplete ESD was 70.3±6.5 years and the mean maximum tumor size was 25.9±15.5 mm. Reasons for performing an incomplete ESD were as follows: accidental perforation or bleeding during ESD (9 patients), lymphatic and/or venous invasion in the ESD specimens (8 patients) and exposed tumor cells in the ESD margins (23 patients; Fig. 1). Radical gastrectomy was performed in 23 patients due to a diagnosis of having exposed tumor cells in the vertical and/or horizontal margins of the ESD specimens. Thirteen patients had exposed tumor cells only in the vertical margin, 6 patients only in the horizontal margin and 4 patients in both margins (Table II). Twenty patients underwent en bloc resection and the remaining 3 patients underwent piecemeal resection. Of 17 lesions with exposed tumor cells in the vertical margins of the ESD specimens, only 3 (17.6%) had residual tumor cells in the corresponding site of the surgically resected specimens. By contrast, of 10 lesions with exposed tumor cells in the horizontal margins of the ESD specimens, 8 (80.0%) had residual tumor cells in the corresponding site of the surgically resected specimens. In the vertical margins of the surgically resected specimens, the length of the exposed tumor in patients with residual tumor cells was 5.7±4.0 mm, which was significantly greater than that in patients without residual tumor cells (1.2±1.6 mm; Table III). In contrast to the vertical margins, no differences were observed in the lengths of the exposed tumors between patients with residual tumor cells in the horizontal margins of the surgically resected specimens and those without. No differences were observed with respect to age, gender, tumor location, tumor depth, lymphatic or venous invasion and lymph node metastases between patients with residual tumor cells in the surgically resected specimens and those without.

In the univariate analysis, no parameter was associated with the incidence of residual tumor cells in the horizontal margins of the surgically resected specimens. On the other hand, the length of the exposed tumor in the vertical margins of the ESD specimens was significantly associated with the incidence of residual tumor cells in the vertical margins of the surgically resected specimens (Table IV). The length of the exposed tumor in the vertical margins of the ESD specimens was found to be the most reliable parameter for distinguishing between the surgically resected specimens that were positive and negative for residual tumor cells in terms of AUROC, although we could not find such differences in the horizontal margins of the ESD specimens. When the cut-off value for the length of the exposed tumor in the vertical margins of ESD specimens was set to >3 mm, the sensitivity, specificity and positive and negative predictive values were 0.67, 0.95, 0.67 and 0.95, respectively (Table V).

Recurrence was not observed in any patient following curative surgery during a mean follow-up period of 36.9 (range 11–70) months.

## Discussion

In Japan, the indications for ER in early gastric cancer patients include well- or moderately differentiated adenocarcinoma restricted to the mucosal layer without ulceration (9,13,14); more recently, these indications have been extended (13,14). The indication for additional gastrectomy following incomplete ER is to remove the residual cancer cells at the site of the ER and/or the potentially metastatic regional lymph nodes. The risks for residual tumors or lymph node metastases following an incomplete ER have been extensively discussed in previous studies (11,15,16). Song *et al* reported that the residual tumor rate in the surgically resected specimens of patients with tumor-positive ER margins was >70% in a Korean multicenter study (17). Residual tumors in the surgically resected specimens have been found in 5.8–63.0% of patients with tumor-positive horizontal margins and in 35–50% of patients with tumor-positive vertical margins in the ESD specimens (11,15,18). These findings are consistent with our results, suggesting that the remaining patients who were pathologically diagnosed with tumor-positive ER margins theoretically did not require radical gastrectomy, except for a potentially high risk of lymph node metastases.

To the best of our knowledge, no study has used the extent of tumor invasion in the ER margins to predict the presence of residual tumor cells in the surgically resected specimens. In this study, we demonstrated that the length of the exposed tumor in the vertical ESD margins was an exclusive parameter that could predict the presence of residual tumor cells in the surgically resected specimens. Only 1 of 14 patients (7.1%) with ≤1 mm of exposed tumor in the vertical ESD margins had residual tumor cells in the surgically resected specimen (Table II). We evaluated the sensitivity, specificity and positive and negative predictive values using serial cut-off values around the inflection points on the receiver operating characteristic (ROC) curve for the length of the exposed tumors in the vertical ESD margins. When the cut-off value was set to >3 mm, the specificity was 0.95. Therefore, patients with exposed tumors >3 mm in length in the vertical ESD margins should be considered as candidates for additional radical gastrectomy. In patients with exposed tumors of ≤3 mm in length in the vertical ESD margins, the optimal treatment should be determined on the basis of risks for surgery and general anesthesia.

Although a tumor-positive horizontal margin is not required for the evaluation of tumor extent, the residual tumor rate was higher in patients with tumor-positive horizontal margins than in those with tumor-positive vertical margins. Indeed, 80% of lesions with exposed tumor cells in the horizontal margins of the ESD specimens had residual tumor cells in the surgically resected specimens. Although repeat ER may not be feasible due to the increased complication rate resulting from scar formation or thinning of the gastric wall following the first ER procedure (10), it appears to be relatively safe in the case of tumor-positive horizontal ER margins compared with the tumor-positive vertical ER margins (19). In this study, the horizontal ER margins were not identified as predictive factors for residual tumors, but the vertical ER margins were. Therefore, additional ER or gastrectomy should be considered when exposed tumors are observed in the horizontal ER margins regardless of the length of the these tumors (11).

As the indications for ER have been extended, the number of patients who undergo incomplete ER should increase. In this study, we did not have a large enough sample size of patients with ER margins positive for tumor cells, which is the limitation of this study, and it is necessary to conduct a multicenter prospective randomized study in order to verify our results.

In conclusion, for early gastric cancer, the measurement of the length of the exposed tumor in the ER margins, especially in the vertical margins, is a simple procedure that is able to determine whether an additional surgical intervention is necessary, except for a potentially high risk of lymph node metastases. Although the indication of additional surgery is generally decided not only by the tumor-positive ER margin but also by other pathological factors, this method may be used to prevent unnecessary surgery in patients with early gastric cancer, especially in high-risk patients for whom general anesthesia is not suitable.

## Figures and Tables

**Table I:** Demographic data of patients who underwent incomplete ESD for early gastric cancer.

Feature	n (%) or mean ± SD
Number	41
Age (years)	70.3±6.5
Gender	
Male	33 (80.5)
Female	8 (19.5)
Tumor depth	
Mucosal invasion	13 (31.7)
SM1	10 (24.4)
SM2	16 (39.0)
MP	2 (4.9)
Tumor location	
U	12 (29.3)
M	9 (22.0)
L	20 (48.8)
Gross type	
Elevated	21 (51.2)
Depressed	20 (48.8)
Maximum tumor size (mm)	25.9±15.5
Histological classification	
Well-differentiated	23 (56.1)
Moderately differentiated	9 (22.0)
Poorly differentiated	2 (4.9)
Papillary	4 (9.8)
Carcinoid	2 (4.9)
Mucinous	1 (2.4)
Lymphatic invasion	
Yes	23 (56.1)
No	18 (43.9)
Venous invasion	
Yes	28 (68.3)
No	13 (31.7)
Lymph node metastasis	
Positive	4 (9.8)
Negative	37 (90.2)
Surgical procedure	
Distal gastrectomy	22 (53.7)
PPG	8 (19.5)
Proximal gastrectomy	6 (14.6)
Total gastrectomy	4 (9.8)
No surgery	1 (2.4)
Open surgery	25 (62.5)
Laparoscopic surgery	15 (37.5)

SM1, submucosal invasion <500 μm; SM2, submucosal invasion ≥500 μm; MP, muscularis propria invasion; U, upper third of the stomach; M, middle third of the stomach; L, lower third of the stomach; PPG, pylorus-preserving gastrectomy.

**Table II:** Clinicopathological findings in patients who underwent radical gastrectomy due to being diagnosed with, or suspected of having, exposed tumor cells in the vertical and/or horizontal margins of the ESD specimens.

								Horizontal margin		Vertical margin			
Patient	Age (years)	Gender	Location	Gross type	Histological type	En bloc resection	Maximum tumor size (mm)	Diagnosis	Exposed tumor length (mm)	Diagnosis	Exposed tumor length (mm)	Tumor depth	Residual tumor in surgically resected specimens
1	67	Male	M	Depressed	Tub1	Yes	10	Positive	2	Positive	8	MP	HM, VM
2	75	Female	L	Elevated	Pap	No	27	Positive	8	Negative	0	Muc I	HM
3	79	Male	L	Depressed	Tub1	No	25	Positive	8	Negative	0	SM1	HM
4	68	Female	L	Depressed	Tub2	Yes	30	Negative	0	Positive	2	SM1	-
5	65	Male	L	Elevated	Tub1	Yes	10	Negative	0	Suspected	0	SM1	-
6	72	Male	L	Elevated	Pap	Yes	26	Positive	14	Negative	0	SM2	-
7	59	Female	M	Elevated	Tub1	Yes	70	Positive	55	Negative	0	Muc I	HM
8	55	Male	M	Depressed	Tub2	Yes	32	Negative	0	Suspected	0	SM1	-
9	63	Female	M	Elevated	Carcinoid	Yes	9	Negative	0	Positive	1	SM2	-
10	69	Male	M	Elevated	Tub1	No	20	Positive	8	Negative	0	Muc I	HM
11	70	Female	L	Elevated	Muc	Yes	38	Positive	10	Positive	8	SM2	HM, VM
12	72	Male	L	Depressed	Tub1	Yes	19	Positive	2	Negative	0	Muc I	HM
13	73	Male	L	Elevated	Tub1	Yes	67	Negative	0	Positive	6	SM2	-
14	70	Male	L	Depressed	Tub2	Yes	16	Negative	0	Suspected	0	SM2	-
15	73	Male	U	Depressed	Tub1	Yes	18	Positive	5	Positive	1	SM1	HM
16	66	Male	L	Elevated	Tub1	Yes	19	Negative	0	Suspected	0	Muc I	-
17	73	Male	M	Depressed	Tub1	Yes	30	Negative	0	Positive	3	SM1	-
18	75	Male	L	Elevated	Por	Yes	8	Negative	0	Positive	3	SM2	-
19	80	Male	U	Depressed	Tub1	Yes	22	Negative	0	Positive	3	SM1	-
20	65	Male	U	Elevated	Tub1	Yes	14	Positive	4	Positive	1	Muc I	VM
21	65	Male	L	Depressed	Tub2	Yes	41	Negative	0	Positive	1	SM2	-
22	61	Male	L	Depressed	Tub2	Yes	16	Negative	0	Positive	2	SM1	-
23	72	Male	U	Elevated	Tub1	Yes	19	Negative	0	Positive	1	Muc I	-

U, upper third of the stomach; M, middle third of the stomach. L, lower third of the stomach; Tub1, well-differentiated adenocarcinoma; Tub2, moderately differentiated adenocarcinoma; Pap, papillary adenocarcinoma; propria invasion. HM, horizontal margin; VM, vertical margin.

**Table III:** Length of the residual tumor in the ESD margins.

A, Exposed tumor length in the horizontal margins of the ESD specimens (n=10).		

	Mean ± SD (mm)	P-value

Surgical specimen in the lateral margin		
Positive for residual tumor cells (n=8)	12.3±17.5	0.8104
Negative for residual tumor cells (n=2)	9.0±7.1	

B, Exposed tumor length in the vertical margins of the ESD specimens (n=17).		

	Mean ± SD (mm)	P-value

Surgical specimen in the vertical margin		
Positive for residual tumor cells (n=3)	5.7±4.0	0.0103
Negative for residual tumor cells (n=14)	1.2±1.6	

ESD, endoscopic submucosal dissection.

**Table IV:** Univariate analysis and the AUROC curve of the factors associated with the incidence of residual tumor cells in the surgically resected specimens.

A, Horizontal margin				

	Hazard Ratio	95% CI	P-value	AUROC

Age, 1-year increments	0.98	0.914–1.046	0.5131	0.656
Gender (male)	1.31	0.339–5.093	0.6930	0.524
Tumor location (U)	1.54	0.325–7.314	0.5860	0.687
Tumor size (≥20.0 mm)	0.92	0.259–3.259	0.8963	0.562
Gross type (elevated)	1.39	0.390–4.936	0.6130	0.548
Tumor depth				
SM2 or deeper	1.22	0.315–4.727	0.7732	0.762
Nodal involvement (compared with N0)				
N1	0.84	0.198–3.598	0.8182	0.562
Lymphatic invasion (yes)	12.00	0.489–294.59	0.1281	0.687
Venous invasion (yes)	1.67	0.537–5.168	0.3763	0.562
Exposed tumor length (mm)	1.02	0.892–1.163	0.7875	0.562

B, Vertical margin				

	Hazard Ratio	95% CI	P-value	AUROC

Age, 1 year increments	1.03	0.845–1.249	0.7872	0.552
Gender (male)	0.60	0.039–9.156	0.7133	0.542
Tumor location (U)	1.67	0.109–25.434	0.7133	0.542
Tumor size (≥20.0 mm)	1.40	0.145–13.569	0.7715	0.542
Gross type (elevated)	0.17	0.013–2.160	0.1704	0.708
Tumor depth				
SM2 or deeper	6.00	0.463–77.753	0.1704	0.708
Nodal involvement (compared with N0)				
N1	0.46	0.096–2.212	0.3324	0.750
Lymphatic invasion (yes)	0.71	0.254–1.994	0.5176	0.708
Venous invasion (yes)	5.00	0.419–59.660	0.2032	0.667
Exposed tumor length (mm)	2.34	1.005–5.429	0.0488	0.865

CI, confidence interval; U, upper third of the stomach; SM2, submucosal invasion ≥500 μm; AUROC, area under the receiver operating characteristic.

**Table V:** The sensitivity, specificity and positive and negative predictive values of being positive for residual tumor cells in the surgically resected specimens according to the length of the exposed tumors in the vertical ESD margins.

Length of exposed tumor (mm)	Sensitivity	Specificity	PPV	NPV
>0	1.00	0.53	0.25	1.00
>1	0.67	0.68	0.25	0.93
>3	0.67	0.95	0.67	0.95
>6	0.67	1.00	1.00	0.95

PPV, positive predictive value; NPV, negative predictive value; ESD, endoscopic submucosal dissection.
